# Dietary restriction after cold snare polypectomy of colorectal polyp for prevention of delayed bleeding

**DOI:** 10.1002/jgh3.12987

**Published:** 2023-10-25

**Authors:** Kentaro Mochida, Fumiaki Ishibashi, Sho Suzuki, Daisuke Saito, Tomohiro Kawakami, Konomi Kobayashi, Mizuki Nagai, Tetsuo Morishita

**Affiliations:** ^1^ Department of Gastroenterology International University of Health and Welfare Ichikawa Hospital Chiba Japan; ^2^ Endoscopy Center Koganei Tsurukame Clinic Tokyo Japan; ^3^ Department of Gastroenterology and Hepatology Kyorin University School of Medicine Tokyo Japan

**Keywords:** adenoma, cold snare polypectomy, colonoscopy, colorectal polyp, dietary restriction

## Abstract

**Background and Aim:**

Cold snare polypectomy (CSP) for small colorectal polyps is a safe technique; however, there is little evidence on whether dietary restriction after CSP is essential. This study aimed to determine whether dietary restriction after CSP is necessary to prevent delayed bleeding.

**Methods:**

This is a randomized, controlled, non‐inferiority trial conducted between November 2021 and March 2022. Patients with non‐pedunculated small colorectal polyps (<10 mm) and who did not take anticoagulants were randomly allocated to two groups: (i) the normal diet (ND) group, and (ii) the low‐residue diet (LRD) group. The ND group was instructed to eat anything after CSP, whereas the LRD group was advised to take LRD for 3 days after CSP. The primary endpoint was the occurrence of delayed major bleeding that needed endoscopic hemostasis.

**Results:**

A total of 193 patients (average 57.5 years old, 51.9% male) were enrolled in the study. Subsequently, 97 and 96 patients were allocated to the ND and LRD group, respectively. The occurrence of delayed major bleeding was 1.0% in the ND group and 2.1% in the LRD group (95% confidence interval [CI]: −4.4% to 2.4%; difference: −1.1%), which showed the non‐inferiority of the ND group. In addition, there was no difference between the two groups with respect to the occurrence of minor delayed bleeding (3.1% and 4.2%, respectively; difference: −1.1% [95% CI: −6.4% to 4.2%]).

**Conclusion:**

Dietary restriction after CSP for low‐bleeding‐risk colorectal polyps is not necessary for the prevention of delayed bleeding (Registration number: UMIN000045669).

## Graphical abstract

This randomized controlled, non‐inferiority trial aimed to determine whether dietary restriction after cold snare polypectomy (CSP) for colorectal polyps is necessary to prevent delayed bleeding. A total of 193 patients were randomly allocated to the low residue diet (LRD) group and the normal diet (ND) group. The primary endpoint, that is, the occurrence of delayed major bleeding, was 1.0% in the ND group and 2.1% in the LRD group (95% CI: −4.4% to –2.4%; difference: –1.1%).

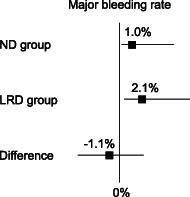

## Introduction

Colorectal cancer (CRC) is the second leading cause of cancer death in the United States and Japan.^1^, ^2^ It has been shown that the removal of all identified adenomatous polyps during colonoscopy improves CRC‐specific mortality.^3^, ^4^ In addition, the risk of interval CRC has been shown to be directly related to the endoscopist's quality indicators, including adenoma detection rate (ADR).^5^, ^6^ In this context, it is recommended that endoscopists should maintain a high ADR.^7^ Thus, endoscopists have become more concerned about their own ADRs and are resecting colorectal polyps more frequently in a single examination.^8^

The technique to resect polyps should be easy, safe, and effective; hence, cold snare polypectomy (CSP) is becoming the first choice for the resection of small polyps.^9^ Several reports have demonstrated increased safety of CSP for small polyps compared to hot snare polypectomy.^10^, ^11^

It was hypothesized that starting diet immediately after the resection of colorectal polyps would increase the risk of delayed bleeding. Retrospective studies have investigated the timing when patients who received endoscopic submucosal dissection (ESD) or endoscopic mucosal resection (EMR) could start eating after treatment.^12^, ^13^ The colorectal ESD/EMR guideline published by the Japanese Society for Gastrointestinal Endoscopy states that the diet should be started with consideration of possible delayed bleeding, but it does not specifically determine when the diet should be initiated.^14^ In particular, CSP, which is safer than EMR or ESD, may not increase the risk of delayed bleeding, even with no diet restriction after treatment. However, there have been no reports on the validity of not restricting the diet after CSP. Therefore, this study aimed to determine whether the absence of dietary restriction after CSP decreases the rate of delayed bleeding compared to dietary restriction with a low‐residue diet (LRD).

## Methods

### Study design

This is a randomized, controlled, open‐label, non‐inferiority trial. The study protocol was approved by the Institutional Review Board (IRB) of Shinjuku Tsurukame Clinic (approval number: 2104) on 9 September 2021, and registered in the University Hospital Medical Information Network Clinical Trials Registry (UMIN‐CTR) (Registration number: UMIN000045669). This study was conducted from November 2021 to March 2022. The study protocol was registered prior to the start of subject enrollment and designed in accordance with the CONSORT statement.

### Subjects

Patients aged 20–80 years with colorectal polyps resected by CSP during colonoscopy were eligible for enrollment in the study. The indication for CSP was defined as colonic polyp less than 10 mm with non‐pedunculated morphology, and lesions not suspected to be cancer. The exclusion criteria were as follows: (i) patients whose other lesion was resected by an energization technique including EMR and hot snare polypectomy (HSP), (ii) those taking anticoagulants, (iii) those with bleeding tendency due to liver cirrhosis or coagulopathy, (iv) those with advanced colorectal cancer, (v) those who did not receive total colonoscopy, and (vi) those with inflammatory bowel disease. Written informed consent was obtained from all study participants prior to starting their examination. The patients were randomly allocated to either the normal diet (ND) group or the LRD group. Randomization was performed by a computer‐generated program and a random number table.

### Intervention

The patients enrolled in this study were not blinded. The patients in the ND group were instructed to eat anything freely after CSP, whereas those in the LRD group were instructed to take LRD for 3 days after CSP. The LRD group was instructed not to eat high‐fiber foods (leafy greens, mushrooms, root vegetables, legumes, etc.), while they were recommended to take rice, noodles, eggs, and yogurt, which are easily digested. Both groups were instructed not to drink alcohol for 1 week.

### Outcomes

The primary endpoint of this study was major delayed bleeding rate until 2 weeks after CSP in the two groups. The secondary endpoints were minor delayed bleeding rate, major and minor delayed bleeding rate according to the polyp size and location of the polyp (the proximal colon and distal colon), and delayed bleeding rate in cases of intraoperative bleeding requiring hemostasis with clips. The predictive factors for major delayed bleeding after CSP were also investigated as the secondary endpoint.

Major delayed bleeding was defined as clinically significant delayed bleeding that needed endoscopic hemostasis. Minor delayed bleeding was defined as slight bleeding that did not require emergency endoscopy. All enrolled patients were asked by phone if they had bleeding 3–5 days after CSP. Patients were also instructed to visit our clinic at 2 weeks after CSP, at which time they were asked if they had any delayed bleeding. In addition, patients were told to call the clinic to report any heavy bleeding before the second‐week visit. Emergency colonoscopy was performed if deemed necessary by the physician. All data related to the outcomes were subsequently recorded and stored in the data center.

### Procedure

All patients underwent bowel preparation with 2 L of polyethylene glycol solution.^15^ If sedation was desired, low‐dose propofol was used unless there were contraindications. Sedation with a combination of pethidine hydrochloride and midazolam was only employed when propofol could not be used owing to allergies or contraindications. All identified polyps during colonoscopy were carefully observed, and lesions that appeared to be adenomatous polyps were indicated for endoscopic resection. The polyps were diagnosed on the basis of their appearance with white‐light imaging and image‐enhanced endoscopy, including narrow‐band imaging and indigo‐carmine splaying.

CSP was performed to resect non‐pedunculated small polyps <10 mm. After CSP, if there was no sign of spontaneous hemostasis after 30 s of observation, endoscopic hemostasis was performed with clipping (EZ Clip, Olympus, Tokyo, Japan). Pedunculated polyps or larger lesions (≥10 mm) were resected through HSP or EMR using SnareMaster Plus (Olympus, Tokyo, Japan). The colonoscopy devices used were CF‐HQ290ZI, PCF‐H290ZI, and CF‐Q260AZI (Olympus). After examination, the endoscopists reported the presence, distribution, and resection of all polyps identified.

### Sample size calculation

The sample size of the non‐inferiority trial required for an adequate comparison of delayed bleeding rate was calculated based on previous data. Delayed bleeding occurred in 2 (0.17%) of 1191 colorectal CSPs <10 mm in size performed at our institution from April 2019 to March 2020, with a low risk of bleeding. A previous study reported post‐CSP bleeding rates of up to 3.8% for high‐risk polyps >10 mm.^16^ Two patients (2.4%) had post‐CSP bleeding for 83 polyps >10 mm during this period at our institution. Because there are no previous studies of interventions similar to ours, we assumed that the risk of delayed bleeding resulting from the intervention in this study was equivalent to the risk of CSP for lesions >10 mm. Assuming a non‐inferiority margin of 3.0%, a delayed bleeding rate of 2.4% in the ND group, and a delayed bleeding rate of 0.17% in the LRD, the number of cases required to obtain a power (1 − *β*) of 0.8 was calculated to be 94 cases per group, with a significance level (*α*) of 0.05. Considering that there would be a 20% dropout after enrollment, a final sample size of 120 cases in each group was determined to be necessary.

### Data analysis

After gathering all the data, an investigator who was not involved in performing colonoscopy and collecting the data compiled and analyzed all the stored data. All statistical analyses and sample size calculations were performed with R 4.0.4.^17^

For the primary endpoint, the two groups were considered clinically equivalent if the 95% confidence interval (95% CI) of the difference in delayed bleeding rates between the two groups was within the non‐inferiority margin, or if the 95% CI of the difference in delayed bleeding rates between the two groups included 0. The average values of the two groups were compared with Student's *t*‐test. The categorical variables were compared with the chi‐square test or Fisher's exact test. Logistic regression analysis was performed to predict whether the absence of dietary restriction after CSP would be a risk for major delayed bleeding, and the odds ratios and 95% CIs were calculated. Statistical significance was set at a *P*‐value <0.05.

This study was conducted according to the guidelines of the Declaration of Helsinki.

## Results

### Background characteristics of patients

**Figure 1:**
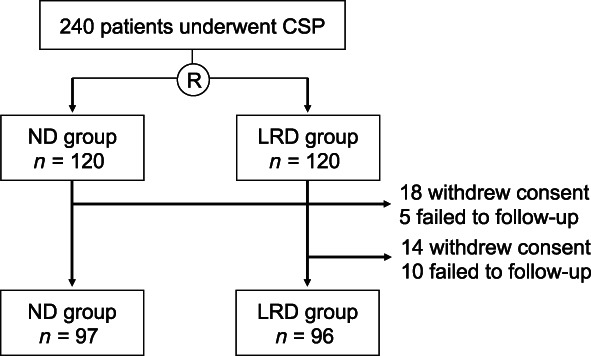
Study flow diagram. A total of 240 patients underwent cold snare polypectomy (CSP) for the resection of colorectal polyps. After randomization, each of the 120 patients was allocated to the normal diet (ND) or low‐residue diet (LRD) group. Eighteen patients withdrew the consent and five patients failed to follow‐up in the ND group. Fourteen patients withdrew the consent and 10 patients failed to follow‐up in the LRD group. Consequently, 97 patients in the ND group and 96 patients in the LRD group were regarded eligible for the comparison.

Initially, there were 120 patients enrolled in each group. After excluding patients who withdrew consent or were not available for follow‐up, 97 patients in the ND group and 96 patients in the LRD group were included in the analysis (Fig. 1). Comparing the background characteristics of the enrolled patients, there was no difference between the two groups in age, sex, body mass index, and presence of any underlying diseases (Table 1). There was also no difference between the two groups in the indication for receiving colonoscopy and the ratio of use of sedative agents. As for the characteristics of treated lesions, there was no difference between the two groups in the number of resected polyps in the two groups (1.5 ± 1.1 *vs* 1.7 ± 1.1, *P* = 0.769), size of resected polyps (4.9 ± 2.1 mm *vs* 4.7 ± 2.1 mm, *P* = 0.821), location where the polyp was resected (40.2% *vs* 42.7%, *P* = 0.821), and ratio of intraoperative bleeding (2.1% *vs* 5.2%, *P* = 0.433).

**Table 1:** Background characteristics

	ND group (*n* = 97)	LRD group (*n* = 96)	*P*‐value
Patient characteristics			
Age (years)	55.9 ± 11.4	58.7 ± 12.5	0.113
Sex (male/female)	51/46	48/48	0.830
Body mass index	23.6 ± 4.0	23.3 ± 3.4	0.508
Underlying disorders			
Cardiovascular disease (%)	8 (9.0)	5 (5.2)	0.579
Diabetes mellitus (%)	8 (9.0)	13 (13.5)	0.342
Chronic liver disease (%)	2 (2.1)	3 (3.1)	0.991
Chronic renal failure (%)	0 (0)	0 (0)	NA
Indication (screening/surveillance)	45/52	55/41	0.170
Sedative agent use (%)	53 (54.6)	52 (54.1)	1.000
Lesion characteristics			
Number of polyps	1.5 ± 1.1	1.7 ± 1.1	0.769
Polyp size (mm)	4.9 ± 2.1	4.7 ± 2.1	0.821
Location (proximal colon) (%)	39 (40.2)	41 (42.7)	0.836
Endoscopic hemostasis after CSP (%)	2 (2.1)	5 (5.2)	0.433

Age, body mass index, number of polyps, and polyp size are expressed as mean ± SD.

CSP, cold snare polypectomy; LRD, low‐residue diet; NA, not applicable; ND, normal diet.

### Primary endpoint

**Figure 2:**
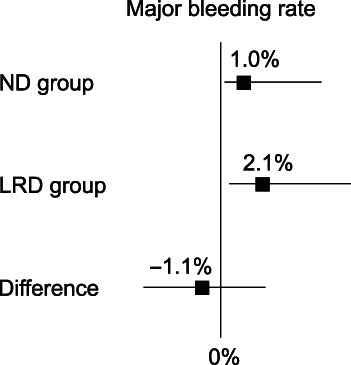
Primary endpoint. The major delayed bleeding rate of the normal diet (ND) group was 1.0% (95% confidence interval [CI]: 0.03–5.6%) and that of the low‐residue diet (LRD) group was 2.1% (95% CI: 0.3–7.3%). The difference between the two groups was −1.1% (95% CI: –4.4% to 2.4%).

The major delayed bleeding rate that needed endoscopic hemostasis was 1.0% (95% CI: 0.03–5.6%) in the ND group and 2.1% (95% CI: 0.3–7.3%) in the LRD group (Fig. 2). The difference between the two groups was −1.1% (95% CI: –4.4 to 2.4%), which revealed non‐inferiority of elimination of diet restriction on the prevention of delayed bleeding after CSP.

### Secondary endpoints

**Figure 3:**
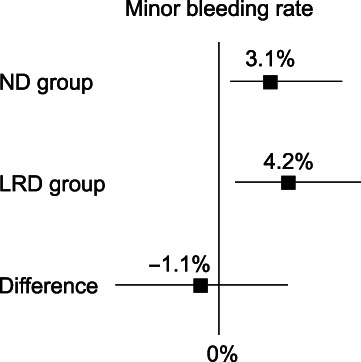
Secondary endpoint. The minor delayed bleeding rate of the normal diet group was 3.1% (95% confidence interval [CI]: 0.64–8.8%) and that of the low‐residue diet group was 4.2% (95% CI 1.1–10.3%). The difference between the two groups was −1.1% (95% CI: –6.4% to 4.2%).

The minor delayed bleeding rate, which did not need endoscopic hemostasis, was 3.1% (95% CI: 0.64–8.8%) in the ND group and 4.2% (95% CI: 1.1–10.3%) in the LRD group (Fig. 3). The difference between the two groups was −1.1% (95% CI: –6.4 to 4.2%). Subgroup analyses in the patients with delayed bleeding showed that there was no difference between the two groups with regard to the patients' background, including age, sex, body mass index, and underlying diseases (Table 2). There was also no difference between the two groups in the lesion characteristics, including polyp size, location of polyp, and the presence of endoscopic hemostasis after CSP. The logistic regression model revealed that ND was not an independent predictor for major delayed bleeding (Table 3).

**Table 2:** Secondary endpoints

	ND group (*n* = 4)	LRD group (*n* = 6)	*P*‐value
Patient characteristics			
Age (years)	58.8 ± 9.3	55.7 ± 7.6	0.620
Sex (male/female)	1/3	2/4	1.000
Body mass index	22.9 ± 4.0	22.4 ± 2.0	0.821
Underlying disorders			
Cardiovascular disease (%)	0 (0)	0 (0)	1.000
Diabetes mellitus (%)	1 (25)	0 (0)	0.400
Chronic liver disease (%)	0 (0)	0 (0)	1.000
Chronic renal failure (%)	0 (0)	0 (0)	1.000
Lesion characteristics			
Number of polyps > 2 (%)	1 (25)	1 (16.7)	1.000
Polyp size > 5 mm (%)	3 (75)	2 (33.3)	0.524
Location (proximal colon) (%)	2 (50)	3 (50)	1.000
Endoscopic hemostasis after CSP (%)	1 (25)	1 (16.7)	1.000

Age and body mass index are expressed as the mean ± SD.

CSP, cold snare polypectomy; LRD, low‐residue diet; ND, normal diet.

**Table 3:** Predictive factors for major delayed bleeding.

	Delayed bleeding (*n* = 10)	No bleeding (*n* = 183)	OR (95% CI)	*P*‐value
Patient characteristics				
Normal diet (%)	4 (40.0)	93 (50.8)	0.78 (0.19–3.2)	0.737
Age > 60 years (%)	3 (30.0)	73 (39.9)	0.87 (0.19–4.0)	0.861
Male sex (%)	3 (30.0)	96 (52.5)	0.51 (0.11–2.3)	0.388
Body mass index > 25 (%)	2 (20.0)	50 (27.3)	0.69 (0.13–3.7)	0.661
Underlying disorders				
Cardiovascular disease (%)	0 (0)	13 (7.1)	NA	0.993
Diabetes mellitus (%)	1 (10.0)	20 (10.9)	1.1 (0.11–11.3)	0.934
Chronic liver disease (%)	0 (0)	5 (2.7)	NA	0.996
Chronic renal failure (%)	0 (0)	0 (0)	NA	NA
Lesion characteristics				
Number of polyps > 2 (%)	5 (50.0)	68 (37.2)	1.6 (0.36–6.8)	0.550
Polyp size > 5 mm (%)	3 (30.0)	77 (42.1)	0.58 (0.12–2.7)	0.483
Location (proximal colon) (%)	6 (30.0)	76 (41.5)	2.0 (0.46–8.2)	0.360
Endoscopic hemostasis after CSP (%)	2 (0)	5 (2.7)	4.1 (0.51–32.2)	0.185

95% CI, 95% confidence interval; CSP, cold snare polypectomy; NA, not applicable; OR, odds ratio.

## Discussion

The ingested meal undergoes a process of digestion and absorption in the stomach and small intestine, and by the time it reaches the cecum, it is almost completely in the liquid state. However, fibrous foods may retain their shape at the time they reach the cecum. Thus, it was hypothesized that hard food residues may cause delayed bleeding if they come into contact with the bottom of the ulcer after endoscopic treatment.

Previous reports that determined the risk factors for delayed bleeding after EMR for colorectal polyps did not analyze whether the timing of food intake may be a risk.^18^, ^19^ Huang *et al*. conducted the non‐inferiority trial to investigate whether dietary restriction after HSP was effective for hospitalized patients. They found no difference between the restricted diet group and the ND group.^20^ However, there has been no report regarding the safety of taking ND after CSP for non‐hospitalized patients. The current study is the first prospective study to investigate the relationship between delayed bleeding after CSP and dietary restriction. The results of this study demonstrate the non‐inferiority of the group without dietary restriction after CSP to the LRD group, with respect to the incidence of delayed bleeding after CSP.

We checked several aspects of this study to validate if it had adequate power. First, as a post hoc detection power analysis, the power back‐calculated using Clamer's V‐statistic was 92.374%. Second, the major delayed bleeding rate observed was 1.0% (95% CI: 0.03–5.6%) in the ND group, which is consistent with the assumed incidence rate (2.4%) used for the sample size calculation. Third, the actual observed difference in event rates between the two groups did not deviate significantly from the non‐inferiority margin. Therefore, we believe that this study design had adequate power to draw the conclusion.

CSP, which has recently become the preferred treatment for small polyps, has been reported to have a lower risk of delayed bleeding than EMR or HSP.^10^, ^21^ One reason for this is that wound healing after CSP is faster than that after EMR.^22^ The major delayed bleeding rate in this study was 1.0% (95% CI: 0.03–5.6%) in the ND group and 2.1% (95% CI: 0.3–7.3%) in the LRD group. A previous study investigating the safety of CSP for polyps at high bleeding risk has reported delayed bleeding rates of 0–3.8%, and the results of this study fall within this range.^16^

Since the study included only non‐pedunculated polyps <10 mm without anticoagulants, strict adherence to the indications is important to keep the delayed bleeding rate low. In recent years, several reports have suggested that CSP can be safely applied to lesions >10 mm, pedunculated polyps, and patients taking antithrombotic drugs, but the safety of CSP for these lesions has not yet been fully evaluated.^9^ In fact, guidelines also state that CSP should be performed only for non‐pedunculated polyps <10 mm.^23^, ^24^ It was not ethically acceptable to include such lesions in this prospective interventional study. In the future, once the efficacy and safety of CSP for such lesions are established, we will expand the scope of this study and verify the results.

The efficacy of prophylactic clipping after EMR for small polyps has been reported to be low.^25^, ^26^ Since CSP is a safer procedure than EMR, prophylactic clipping is expected to be unnecessary even when CSP is performed on small lesions. In the present study, clipping was performed on the resected edge only, when the bleeding did not stop spontaneously after CSP. Proximal colon lesions have been reported to have a high risk for clinically significant delayed bleeding after EMR.^27^ Prophylactic clipping has also been reported to be effective for large lesions of the proximal colon.^28^ However, the effectiveness of prophylactic clipping for small lesions, as well as for polyps in the distal colon, has been shown to be low.^26^ The results of the secondary endpoint analysis of this study also showed no association between whether the proximal colon was involved in the cases of delayed bleeding.

There was no difference in the background of bleeding cases between the ND and LRD groups; however, the power of this study might not have been enough to identify the risk factor for bleeding because there were only 10 cases of delayed bleeding recorded. In addition, the logistic regression model showed that there was no significant predictor for the delayed bleeding after CSP. Since CSP is an established and safe surgical method,^10^ delayed bleeding is completely incidental and its occurrence can be difficult to predict as long as the indications for CSP are adhered to.

This study has several limitations. First, it was conducted at a single institution in Japan. The study participants were all Japanese, thus the generalizability of results might be low. In the future, it is recommended that similar trials be conducted in other countries. Second, the sample size should have been as small as possible because the study aimed to show non‐inferiority of the intervention group. As a result, the occurrence of clinically significant delayed bleeding was low; thus, the sub‐analysis on the background characteristics of these delayed bleeding cases involved a small number. Future larger prospective cohort studies would reveal the exact characteristics of clinically significant delayed bleeding cases in patients without dietary restriction after CSP. Third, there were dropout cases despite the short‐term follow‐up. The reasons for this was that a certain number of patients had been instructed to visit the clinic exactly 2 weeks later but were seen 3 weeks later. In addition, a significant number of patients withdrew their consent after allocation because the dietary restriction instructions were not to their preference. However, we believe that the study is valid because the sample size was maintained even after dropout cases were excluded.

In conclusion, the non‐inferiority randomized controlled trial revealed that dietary restriction after CSP for colorectal polyps of low bleeding risk is not necessary for the prevention of clinically significant delayed bleeding.

## Patient consent

Written consent was obtained from all enrolled subject.

## Data Availability

All data generated or analyzed during this study are included in this article. Further enquiries can be directed to the corresponding author.
